# A Narrative Review of Current Knowledge on Cutaneous Melanoma

**DOI:** 10.3390/clinpract14010018

**Published:** 2024-01-26

**Authors:** Bogdan Marian Caraban, Mariana Aschie, Mariana Deacu, Georgeta Camelia Cozaru, Mihaela Butcaru Pundiche, Cristian Ionut Orasanu, Raluca Ioana Voda

**Affiliations:** 1 Clinical Department of Plastic Surgery, Microsurgery–Reconstructive, “Sf. Apostol Andrei” Emergency County Hospital, 900591 Constanta, Romania; 2 Faculty of Medicine, “Ovidius” University of Constanta, 900470 Constanta, Romania; 3 Clinical Service of Pathology, Departments of Pathology, “Sf. Apostol Andrei” Emergency County Hospital, 900591 Constanta, Romania; 4 Academy of Medical Sciences of Romania, 030171 Bucharest, Romania; 5 The Romanian Academy of Scientists, 030167 Bucharest, Romania; 6 Center for Research and Development of the Morphological and Genetic Studies of Malignant Pathology (CEDMOG), “Ovidius” University of Constanta, 900591 Constanta, Romania; 7 Clinical Service of Pathology, Departments of Genetics, “Sf. Apostol Andrei” Emergency County Hospital, 900591 Constanta, Romania; 8 Clinical Department of General Surgery, “Sf. Apostol Andrei” Emergency County Hospital, 900591 Constanta, Romania

**Keywords:** clinical trials, cutaneous melanoma, epidemiology, immune checkpoint inhibitors, risk factors, targeted therapy

## Abstract

Cutaneous melanoma is a public health problem. Efforts to reduce its incidence have failed, as it continues to increase. In recent years, many risk factors have been identified. Numerous diagnostic systems exist that greatly assist in early clinical diagnosis. The histopathological aspect illustrates the grim nature of these cancers. Currently, pathogenic pathways and the tumor microclimate are key to the development of therapeutic methods. Revolutionary therapies like targeted therapy and immune checkpoint inhibitors are starting to replace traditional therapeutic methods. Targeted therapy aims at a specific molecule in the pathogenic chain to block it, stopping cell growth and dissemination. The main function of immune checkpoint inhibitors is to boost cellular immunity in order to combat cancer cells. Unfortunately, these therapies have different rates of effectiveness and side effects, and cannot be applied to all patients. These shortcomings are the basis of increased incidence and mortality rates. This study covers all stages of the evolutionary sequence of melanoma. With all these data in front of us, we see the need for new research efforts directed at therapies that will bring greater benefits in terms of patient survival and prognosis, with fewer adverse effects.

## 1. Introduction

Despite advances in the field of research, skin melanoma remains a worrisome pathology for health [1]. Efforts to reduce incidence continue to be ineffective, as rates keep increasing [2]. Incidence rates vary across continents: there are <0.5 cases/100,000 in Asia, 1/100,000 in Africa, 13.2/100,000 in Europe, 21.6/100,000 in the USA, and 48/100,000 in Australia. However, on a global scale, the number of cases is steadily rising by 2.6% every year [3,4]. Melanoma is the twelfth most common cancer globally, with higher rates in Europe (seventh) and the USA (fifth) [1,4]. The age-standardized incidence rate observes similar proportions of 3/100,000 women and 3.8 per 100,000 men [5].

Over the years, tremendous efforts have been implemented to decrease the number of cases through primary and secondary prevention programs. Primary prevention consists of avoiding the main risk factor attributed to melanoma’s development: exposure to ultraviolet rays (UV) [1,6]. Worldwide, there have been campaigns to raise awareness of the risk of developing melanomas due to exposure to ultraviolet rays. After observing the favorable outcomes of the 1980s campaign in Australia, other countries like the USA embraced this trend as well, exemplified by “The Surgeon General’s Call to Action to Prevent Skin Cancer” [1]. The United States Preventive Services Task Force strongly advises against sunlight exposure and the use of tanning beds. They also recommend using sunscreen with a sun protection factor of ≥15, and wearing sunglasses, hats, and sun-protective clothing. Also, behavioral counseling is offered to all people aged 6 months and up. These measures involve collaborating with the US Food and Drug Administration to provide information and guidance on sun protection lotions, partnering with the Environmental Protection Agency to offer information, widgets, and weather warning smartphone applications, and working with the Community Preventive Services Task Force to develop policies aimed at promoting preventive behavior [6]. In Germany, the national skin cancer screening program, covered by insurance, has been in action since 2008. Thus, dermatologists and general practitioners must visually examine the entire skin of the patient [7].

Secondary prevention refers to any method that aims to identify high-risk populations, and screening strategies [1]. Secondary prevention is based on the early identification and treatment of pre-lethal injuries. Every person should be educated on self-examination and how to identify signs of concern, giving them access to a healthcare system that can diagnose melanoma early and provide the necessary treatment. Detection programs have been tried in Australia, Belgium, France, Germany, and the USA. The conclusion of the screening programs was a beneficial one, but the evidence was not strong [8]. However, the United States Preventive Services Task Force did not identify sufficient evidence to recommend routine skin screening, even if screening by total body examination of the high-risk population is safe, cost-effective, and efficient (5.2 quality-adjusted life years per 1000 people screened annually). These aspects can lead to overdiagnosis and a potential increase in therapeutic costs with a limited mortality benefit [9]. In this regard, new technologies are being developed to help dermatologists and primary care physicians. They can rely on artificial intelligence, with the most notable examples being multispectral digital skin lesion analysis, image-analysis software, and total body mole mapping [8,9].

Even if the prevention results were favorable (identifying cases at an early stage or reducing the frequency in certain population groups), they did not bring significant improvements, and the frequency of melanomas increased year by year [1,7]. There are undeniable advantages to early detection. This approach minimizes disfigurement by reducing the size and extent of surgical excision, mitigates adverse effects caused by systemic therapy, potentially saves the lives of patients who may not benefit from advanced systemic treatments, and lowers overall healthcare costs [10]. Another strength of early detection resides in the association with patient survival. Melanomas limited to the epidermis (in situ) pose no risk of death, and the likelihood of metastasis is low for thin melanomas [11]. The issue of overdiagnosis is a significant problem, as it does not bring any benefits to the patients. Instead, it can cause them to suffer as a result of both the diagnosis itself and the treatment they will undergo [12]. Overdiagnosis occurs when a tumor is diagnosed as malignant, but would not actually lead to the patient’s death if left untreated. In Australia, a study found that overdiagnosis occurred in 54% of melanoma cases in women and 58% of melanoma cases in men. Thus, although useful, early detection programs are less than 100% effective. This means that for every lesion diagnosed as melanoma, there are other undiagnosed melanocytic lesions [13]. In a study conducted by Kurtansky NR et al., which analyzed nine registries of the Surveillance, Epidemiology, and End Result Program, the authors identified evidence (discrepancies between the relative increase in incidence compared to mortality) that suggested overdiagnosis, especially in middle-aged people and young women [12]. This overdiagnosis not only harms the patient but also the health system through increased costs and use of resources. Unfortunately, it is difficult to determine which melanomas are overdiagnosed or overtreated. Due to this challenge, doctors face significant difficulty in determining the precise adjustments needed in their practice to enhance the overall well-being of their patients [14]. So far, one way to prevent overdiagnosis is to include histopathological parameters like tumor thickness, ulceration, invasion, or mitotic rate in the report. In advanced stages, molecular biology examinations are added [15].

Primary and secondary prevention, along with new therapies, have proven their effectiveness in terms of mortality. In the USA, the mortality rate had an initial evolution of a slight increase—despite a solid prevention program—but after the new treatments were initiated, the mortality rate was a decreasing one [16]. In Europe, mortality differs between regions, with a maximum rate of 3.2:100,000 inhabitants in Norway and 1:100,000 inhabitants in Romania. This aspect represents a paradox given that Northern European countries benefit from much more extensive secondary prevention programs than Central or Eastern European countries [4]. Contrary to these measures, the International Agency for Research on Cancer estimates that the mortality of cases diagnosed with melanoma will increase by 56.92% until 2040 [17].

Without underestimating the role of prevention, studies have shown that the contribution of new immunological therapies is superior [16,18]. In this regard, the most important therapies are represented by immune checkpoint inhibitors (ICIs): anti-CTLA-4 monoclonal antibodies and anti-PD-1 monoclonal antibodies [19,20]. Anti-CTLA-4 medications act by directly inhibiting CTLA-4 with CD80/CD86 ligands, which causes CD28 co-stimulation and T-lymphocyte activation. The immunosuppressive effect in melanoma is partially mediated by Treg recruitment (highly expressed CTLA-4). Anti-CTLA-4 medication causes a reduction in tumor infiltration and circulating Tregs after therapy. In contrast to the modulation of immune function during the initial phase of T-lymphocyte activation, anti-PD-1 therapy functions by halting cell activity during the effector phase. This consists of reducing the number of phenotypically exhausted cytotoxic CD8+ lymphocytes. Also, a response to anti-PD-1 drug treatment is dependent on a T lymphocyte response in the tumor microclimate [21]. Normally, PD-1 binding inhibits the proliferation of T lymphocytes, reduces their overexpression, and inhibits the production of (IFN-γ), tumor necrosis factor-α, and IL-2. Thus, anti-PD-1 medication acts on these immune checkpoints, restoring or increasing the antitumor immune response to achieve tumor regression [22]. The advantages of these therapies consist of the extension of their increased efficiency to metastatic melanomas, or those with a high risk of recurrence [20]. These therapies prove their effectiveness in the advanced stages where surgical excision has limited curative potential. Thus, until stage III, surgery is the first line of treatment [19].

This review provides an in-depth analysis of cutaneous melanoma, highlighting key risk factors, clinical and histopathological features, and the primary mechanisms that shape current treatment approaches. Furthermore, this study aims to provide a comprehensive and detailed analysis of the development process of this life-threatening disease, with the goal of offering valuable insights into the condition and its treatment. In addition, by thoroughly explaining existing therapies and ongoing clinical trials, we aim to highlight the critical significance of and urgent necessity for the advancement of novel therapeutic approaches that offer fewer side effects and improved efficacy. Thus, we want this research to serve as a solid foundation for future study perspectives.

## 2. Cutaneous Melanoma

### 2.1. Risk Factors

Numerous factors have been discovered to have a connection to the development of melanoma over time. To better understand this topic, these factors can be categorized as either environmental or modifiable factors, as well as non-modifiable factors [2,23].

The most important risk factor is exposure to UV radiation. Ultraviolet radiation specifically targets the process of DNA replication. People are exposed 20–40 times more to UVA than UVB; however, UVB waves are 1000 times more toxic [16]. Intermittent exposure to the sun has a 2.35-times-higher relative risk than chronic exposure. Also, a history of sunburn has a negative impact, being associated with the development of melanoma [1,24]. It should be noted that the use of UV radiation also occurs in medical treatments. Certain inflammatory skin conditions are treated with psolaren and ultraviolet A (PUVA). The risk of developing melanoma is increased after 250 sessions [2]. Obtaining cosmetic effects through exposure to artificial UV radiation is not without its risks. In tanning sessions, the level of exposure is significantly higher compared to outdoor activities [25]. Thus, the risk of developing melanoma is 20–75% higher [24,25].

UVB rays act on the skin through the formation of cyclobutane pyrimidine dimers (CPDs) (75%) and pyrimidine–pyrimidone (6-4) photoproducts (6-4PP) (25%) [26,27]. CPDs lead to the substitution of cytosine bases with thymine bases at the DNA level. These changes reflect the genetic imprint of UVB radiation. Both UVA and UVB radiation cause CPD to form, leading to the production of reactive oxygen and nitrogen species. UVA rays are more potent in providing oxidative compounds at the skin level. In addition, oxidative reactions can cause damage such as single-strand breaks and the oxidation of DNA bases (formation of 8-oxo-7,8-dihydro-2′-deoxyguanosine) [26]. In addition to altering DNA integrity, oxidative stress plays a crucial role in modifying the transcriptional profile and the proteins responsible for the dysregulation of numerous oncogenes and tumor suppressor genes [28]. Another effect of reactive oxygen species consists of activating the expression of matrix metalloproteinases. They produce the degradation of collagen fibers, generating the wrinkled and aged appearance of the skin. Another mechanism of UV rays targets pigmented melanocytes and consists of the activation of nitric oxide synthase, with the generation of nitric oxide radicals, NADPH oxidases, and superoxide anion radicals. The interaction between these radicals leads to the formation of peroxynitrite, a powerful oxidizing agent that triggers the excitation of melanin electrons to a heightened energy state, thus influencing the oxidation of melanin [27].

Another environmental factor worth mentioning is the use of herbicides. Melanomas developed following the use of herbicides for personal (at home) or industrial (exposed workers) purposes are rare. They are especially responsible for the development of acral melanomas (on the palms and soles of the feet) [29]. Oxidative stress caused by the generation of reactive oxygen species is considered one of the primary mechanisms behind the development of cancer. In this case, dysfunction manifests at the mitochondrial level, leading to an intervention that halts the G0/G1 cell cycle, ultimately resulting in an elevation of programmed cell death. Moreover, certain herbicides have the potential to impact the regulation of p53 expression, which involves the activation of the p53 protein in response to oxidative stress and DNA damage [29,30].

The most important unmodifiable factors are family history, race, skin type, age, gender, immunosuppression, presence of nevi, etc. [2,16,31].

About 15% of people with melanomas have a family history of the disease. The most involved gene is CDKN2A. It plays a crucial role in the development of atypical moles and superficial spreading melanoma. Gene mutation occurs in sporadic cases of melanomas, in addition to the hereditary component [32]. The second gene with an increased risk of hereditary melanoma is CDK4. The pathogenic pathway is common with CDKN2A, presenting the same consequences. Until now, only a few familial cases with CDK4 mutation have been reported (<1.4%) [33]. Other genes with high penetrance that have been identified, but that are responsible for less than 1% of hereditary cutaneous melanomas, are the following: BAP1, POT1, ACD, TERT, TERF2IP, and BRCA2 [1,32,33]. Another autosomal recessive inherited condition is represented by xeroderma pigmentosum. This disease causes increased sun sensitivity, leading to 20% of patients developing melanoma.

Race or ethnicity plays an important role in the development of skin melanomas. This characteristic is also evident in the higher frequencies found in Caucasian populations and lower frequencies in African American and Hispanic populations [2]. Caucasians are approximately 2.4% more likely to develop melanoma [19]. Variation in skin color is primarily caused by different types of melanin and, secondarily, by the presence of oxyhemoglobin, deoxyhemoglobin, and carotenoids at the skin surface [19,34]. Populations with dark skin have a higher eumelanin (black melanin) content. This has a greater capacity to absorb ultraviolet radiation and eliminate free radicals, having a protective role. Populations with light skin have an increased content of pheomelanin (red melanin). This, following stimulation, reduces glutathione deposits through oxidative processes, causing genetic instability and DNA damage [35,36].

Certain dermoscopic peculiarities can be added to the previously presented differences regarding race or ethnicity. In Caucasian and Hispanic populations, the most common form is superficial melanoma. Among Caucasian populations, melanoma in situ often displays irregular areas of hyperpigmentation, atypical networks, and gray structures more commonly than other characteristics. Similarly, in the case of in situ and invasive melanoma, irregular blotches, atypical networks, and gray or blue structures are characteristic. These aspects are also found in the Hispanic population. Moreover, thick melanomas often exhibit the presence of a blue-white veil, white shiny structures, and milky red areas. Melanomas on the trunk and limbs in Asian individuals exhibit a distinctive and complex array of characteristics, including an asymmetrical and colorful pattern, blotches, a blue-white veil, atypical pigment networks, irregular peripheral streaks, atypical vascular patterns, ulceration, atypical dots/globules, bright white lines, and regression structures. In Black and African populations, a common occurrence is in the palmoplantar location, which exhibits a parallel ridge pattern with a perilesional hypomelanotic halo. The most studied melanomas of the four skin types are acral lentiginous melanomas of the sole. They have common characteristics, regardless of race, represented by a parallel ridge pattern in the palmoplantar areas [37].

The previously stated aspects are closely related to the phototypes of the skin. A person with a phenotype characterized by decreased melanin production (such as light skin and blue eyes) carries a higher risk of melanoma than someone with dark skin. The reason behind this is that individuals with dark skin have a higher concentration of melanin, specifically eumelanin, which is found in the outermost layers of the skin. UVB radiation causes damage to DNA in the outer layers of the skin, but not in the basal cells of the basal layer [31,38]. It is important to note that being able to tan still acts as a protective factor against melanoma [31].

Advanced age and gender are also risk factors involved in the development of melanoma. The female sex is frequently affected until around the age of 40. For men, the frequency of melanoma triples in comparison to women from the age of 75 onwards. Men have a 1.5 times higher risk of developing melanoma compared to women [19,31]. These aspects serve to confirm that the varying distribution according to sex is not solely determined by the cumulative impact of UV radiation [39]. So far, the disparity between the genders in motivation is thought to stem from immune homeostasis, which results from the inactivation of one of the two X chromosomes. This hypothesis supports a better ability to neutralize oxidative stress. While males may have a larger number of natural killer cells, it is the female sex that benefits from their superior functionality, thanks to the heightened efficiency of antigen-presenting cells [40,41].

An increased risk of developing melanomas is found in immunocompromised patients. A group of immunocompromised patients is represented by those who have benefited from organ transplantation. The occurrence of this phenomenon is particularly notable in kidney and liver transplants, with a relative risk ranging from 1.8 to 8 [42]. Although melanoma is not common among HIV-infected patients, they still face certain risks. In these cases, the most common types of skin cancer observed are squamous cell carcinoma and basal cell carcinoma [43]. Chronic lymphocytic leukemia significantly weakens the immune system, leading to a two-to-four-times higher risk of developing melanoma [44]. Likewise, in the case of non-Hodgkin’s lymphomas, the relative risk is increased by 1.6 times [45].

Around 25% of skin melanomas come from the level of pigment nevus. Transformation occurs at the trunk and proximal extremities, areas not affected by chronic sun damage [46]. The majority of pigmented nevi remain unchanged or disappear throughout one’s life. Only a small percentage of them (5%) require careful monitoring [47]. The risk of developing cancer is higher when there are many pigmented nevi, particularly in areas of the skin that are occasionally exposed to the sun [47,48]. The estimated lifetime risk is 1:11,000 for women and 1:3000 for men [46]. The initial phase of the development of cancer involves the mutation of the BRAF V600E gene, leading to the subsequent inactivation of PTEN. From a clinical standpoint, the emergence of melanoma on a nevus follows a distinct pattern: the patient is typically young, exhibiting a heightened presence of nevi, particularly on the torso where sun exposure is sporadic. The most prevalent type is superficial spreading melanoma, which histologically exhibits a shallow Breslow depth, regression features, and a lack of ulceration [49].

Obesity, smoking, and alcohol consumption do not significantly affect the risk of developing melanoma [50,51,52,53]. It has been observed that obese people respond better to targeted or immunological therapies, but the mechanism still unclear [54]. Also, until now, researchers have not identified any association between increased body weight and melanoma. Furthermore, Roccuzzo G et al. conducted a thorough analysis, which revealed that body mass index cannot be used as a reliable indicator for predicting the survival outcomes (such as overall survival and progression-free survival) of patients being treated with immune checkpoint inhibitors [55]. Regarding smoking, studies have revealed that there is no increase in the risk of melanoma. Actually, men have a lower risk [51,56]. While studies have indicated a moderate risk of developing melanomas with alcohol consumption, a direct cause-and-effect relationship between the two has yet to be confirmed. Additionally, further research is necessary to examine the correlation between alcohol consumption, exposure to UV radiation, the phenotype characteristics, and the precise amount of alcohol consumed [52,53].

### 2.2. General Clinical–Histopathological Aspects

Over the years, there have been notable achievements in developing clinical systems for the detection of skin melanomas [57,58,59,60]. The widely adopted system is the American ABCD(E) system, which originated in 1985 [57,58]. Each letter represents an abbreviation for the following: asymmetry, borders, color, diameter, and elevation or evolution. Limitations include the inability to use it in lesions with diameters below 5 mm, amelanotic melanomas, or nodular melanomas, which do not exhibit color heterogeneity or irregular edges [57]. The sensitivity of this test is between 82.6 and 92.8% and the specificity is 70–91.2% [61].

The Glasgow system uses three major criteria (shape, color, change in size) and four minor criteria (diameter ≥ 7 mm, change sensory, crusting or bleeding, and the presence of signs of inflammation) [58].

Dermatoscope examination is the most accessible non-invasive diagnostic technique, as well as the most reliable. The dermatoscopic criteria for diagnosis are represented by an atypical pigment network, irregular globules or dots, irregular streaks, irregular pigmentation, regression structures, a blue-white veil, and vascular patterns [58,62].

Clinical diagnostic systems combine clinical evaluation with dermatoscopy techniques. The Menzies method involves identifying both negative aspects (such as lesion symmetry and the presence of a single color) and at least one positive aspect (like the presence of a blue-white veil, multiple brown dots, pseudopods, radial streaming, scar-like depigmentation, black dots or globules, multiple colors, multiple blue-grey dots, or a broadened network) [59]. The test has a sensitivity of 85.7% and a specificity of 71.1% [61].

The CASH algorithm suggests a melanoma if the sum of the constitutive elements is ≥8. Each point is assigned to a color (blue, white, red, black, dark brown, light brown) and to a homogeneity (polymorphous blood vessels, blotches, regression structures, blue-white veil, pseudopods, globules, atypical network). The scoring system evaluates symmetry and architectural organization, ranging from 0 to 2. It considers biaxial asymmetry as 2, monoaxial symmetry as 1, and biaxial symmetry as 0, while architectural disorganization is rated as 2 for marked, 1 for moderate, and 0 for none/mild [60,63]. The test has a sensitivity of 68% and a specificity of 98% [61].

The seven-point scale is another early clinical diagnosis method. Melanoma is suspected when the sum of the constituent elements is ≥3. Major criteria are scored with two points and include atypical pigmentation, atypical patterns, and a blue-white veil. The minor criteria are marked with one point and include irregular streaks, irregular pigmentation, irregular globules or spots, and areas of regression [59,64]. The sensitivity of this test is 83.6% and the specificity is 71.5% [61].

The three-point checklist is a simplified method composed of the following: asymmetry in color and/or structure in one or two axes; any blue and/or white structure in the lesion, and a pigmented network with thickened lines and irregular distribution [65]. The sensitivity of this test is 91% and the specificity is 71.9% [61].

All these data on detection algorithms are summarized in Table 1.

Last but not least, an element that attracts attention is the sign of an ugly duckling. That is, the pigmented lesion suspected to be melanoma differs from the neighboring pigmented lesions [58].

Upon initial inspection, the lesions appear as macules, papules, nodules, or plaques, with varying sizes, asymmetry, irregular edges, and color variations [66,67]. In histopathology, it is essential to report the size of the excised skin in three dimensions, the dimensions of the lesion, the uniformity of pigmentation, the lesion’s edges, the presence of nodules, and the distances from the surgical sections [57].

The first step in the definitive diagnosis of cutaneous melanomas may consist of a biopsy of the lesion. The recommended technique is excisional biopsy, which offers the highest accuracy according to the Breslow index and histopathological diagnosis [68]. In the case of tumors too large for primary excision, lesions in cosmetically sensitive areas, or on the palms, soles, or fingers, a partial biopsy is performed [69]. The disadvantage of shave biopsy is that it can underestimate the Breslow thickness. Once melanoma is diagnosed after a biopsy and the entire lesion is removed, research has demonstrated that 5-year survival rates are comparable regardless of whether a shave biopsy, punch biopsy, or excisional biopsy is performed [68].

The most common tumor entities, depending on growth patterns, are superficial spreading melanoma, nodular melanoma, lentigo maligna melanoma, and acral melanoma (Table 2) [66].

**Figure 1:**
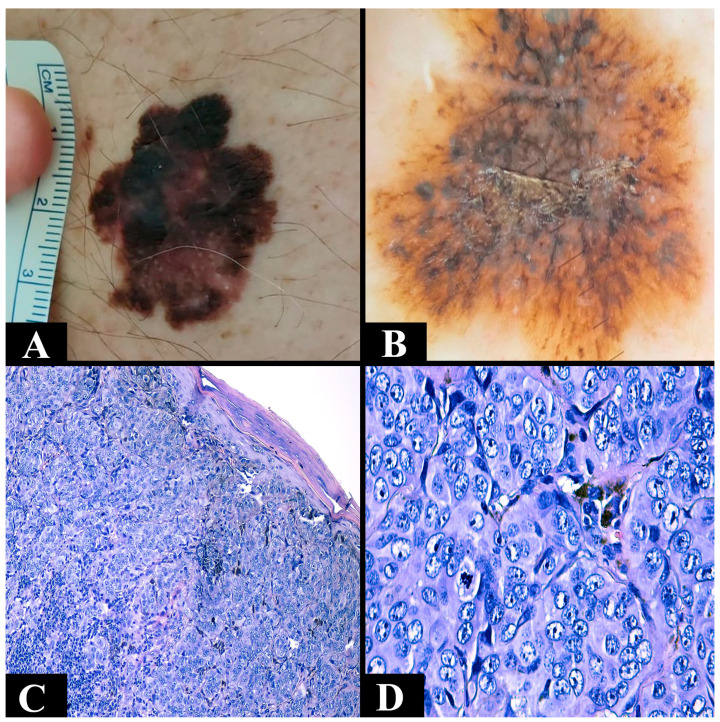
(**A**) Clinical aspect of a superficial spreading melanoma asymmetric (irregular edges and brown-black with a maximum diameter of 3 cm). (**B**) Dermoscopic aspect of a superficial spreading melanoma with irregular edges with an atypical pigmentation network (dark brown and with irregular black dots). (**C**) Microscopic appearance of a nodular melanoma composed of nests of epithelioid atypical melanocytes with a nodular disposition (Breslow 1 mm) with epidermal consumption (hematoxylin–eosin staining, Ob. ×100). (**D**) Microscopic view of nests of atypical melanocytic cells from a nodular melanoma in which cellular pleomorphism and atypical mitosis can be observed (hematoxylin–eosin staining, Ob. ×400).

The most common type is superficial spreading melanoma (Figure 1A,B). It particularly impacts the Caucasian population [75]. The average age of diagnosis is 51 years. It can be located at any level (trunk or extremities) except the palms and soles [66,67]. It can develop de novo or based on a pigmented nevus [66]. During its initial stage, the growth of the lesion is slow and progresses radially. It appears as a brown-to-black macule, measuring less than 5 mm, with irregular edges. The lesion is confined to the epidermis or may extend focally into the papillary dermis. During the vertical growth phase, the rapid development of a nodule or a papule occurs, which may potentially ulcerate [66,75]. Histopathologically, a stage of melanoma in situ is evident, showing a pagetoid extension of malignant melanocytes along the epidermis. The initial invasive stage involves the spread of malignant cells in the superficial dermis [76]. The specific architecture consists of asymmetry, nests of melanocytes of various sizes and shapes, with the absence of maturation in the dermis. It is also associated with different degrees of solar elastosis [77].

The second most common type is nodular melanoma (Figure 1C,D). It is located at the level of the head, neck, or trunk [66,75]. The average age is 56 years [66,67]. Like the previous one, it can develop de novo or based on a pigmented nevus [66]. It has a short radial growth phase that clinically corresponds to a nodular, red or black, well-defined, uniformly colored lesion. The phase of vertical growth is fast and early, meaning that it is often diagnosed in advanced stages [66,75]. Microscopically, it is characterized by malignant melanocytes, frequently of an epithelioid appearance, arranged in the form of cohesive aggregates at the level of the dermis. The epidermal component is reduced to rare cells or nests [70].

Lentigo maligna melanoma represents less than 15% of melanomas. It is located on surfaces chronically exposed to UV rays (nose, cheeks, scalp, ears, etc.) [66,75]. Often, it appears in elderly people after 61 years [66,67]. The precursor lesion is represented by malignant lentigo. Over many years, it progresses to become a macular lesion of different shapes and sizes, featuring irregular, indented edges and brown-black pigment [66,75]. This lesion goes through a phase of radial growth lasting for years in which small, atypical epithelioid melanocytes are observed located at the level of the dermoepidermal junction. Occasionally, an extension to the skin appendages can be seen [75,78]. The progression of a lentigo maligna to a lentigo maligna melanoma occurs in over 50% of cases, and the transition period can be over 35 years [79]. Infiltration of the papillary dermis with at least one malignant cell is sufficient to be considered lentigo maligna melanoma [76]. The classic appearance is represented by small, discohesive cells, with cytoplasm with artifactual retraction and a large, angular nuclei. A solar elastosis of increased intensity and epidermal atrophy are also associated [78].

The rarest form of melanoma encountered is acral melanoma. It is located at the levels of the soles of the feet, palms, or nail apparatus [66,75]. Like the previous subtype, it appears in elderly people after 61 years [66,67]. It develops for months or years in the form of a macule, is pigmented, of variable size with irregular edges, and is less often in the form of warts or nodules [66,75]. Microscopically, in the early stages, architectural changes with the appearance of the coalescence of atypical melanocytes in nests are observed, with a regular distribution and extension of melanocytes in the superficial layers of the epidermis. The cells have a dendritic-like cytoplasm (they develop extensions that form around the basal cells). The nuclei have a vertical arrangement, and are large, hyperchromatic, and angular [80]. In the advanced stages, the architecture is made of single melanocytes or nests of discohesive melanocytes, and is poorly circumscribed. Cellularity shows an asymmetric distribution, with variable distances between cells and a more prominent pagetoid extension. The pigment is arranged in extended areas of the corneous layer. The cells show the same atypical changes previously described [81]. This type of melanoma is not only significant due to its location, but also due to its aggressiveness, having a recurrence rate 2–5 times higher than the others. This is due either to its pathogenic aspects or to the fact that they are diagnosed late [82].

In all four subtypes, the histopathological report must include the diagnosis and the clinicopathological subtype, Breslow depth (measured in millimeters, at a right angle, from the level of the upper surface of the granular layer to the maximum thickness of the tumor), the presence or absence of ulceration, the number of mitoses per mm2 measured in hotspots, the presence or absence of microsatellites at a distance of at least 0.3 cm from the tumor, and the distances from the surgical margins. The growth phase, the presence or absence of regression, the presence or absence of tumor-infiltrating lymphocytes, and lymphatic, vascular, and perineural invasions should also be mentioned [71,83,84]. According to the latest criteria, the level of invasion proposed by Clark in 1966 is no longer mandatory [71,85].

Another important factor to consider is the role of the sentinel lymph node (SLN). The process involves conducting either preoperative or intraoperative lymphatic mapping, followed by the selective removal of the first lymph node in the regional basin [86]. Lesions in the upper extremity drain into the axillary basin, while those in the lower extremity drain into the inguinal basin. Trunk lesions can drain into both basins. Lesions on the head and neck can drain into multiple basins (several satellite nodules are often found), even on the contralateral side [87].

Sentinel lymph node biopsy is a controversial subject, but its prognostic value is immeasurable. Lesions with a thickness greater than 0.75 mm or that exhibit ulceration have a risk of >5% for the development of metastasis in the SLN [88]. The sentinel lymph node is the best predictor for disease recurrence and melanoma-specific death [89]. The guidelines recommend SLN biopsy for melanoma patients in stages >T1b and those with stage T1a with high-risk aspects (ulceration or increased mitotic rate) [86]. Currently, patients with SLN have a diverse array of therapeutic options available to them. These options include surveillance, completing lymph node dissection, and utilizing adjuvant therapy with immune checkpoint inhibitors and targeted therapy [88]. However, in patients under the age of 40, sentinel node biopsy has a low specificity in predicting mortality. Therefore, obtaining a positive outcome could potentially lead to unnecessary treatment, putting patients at risk of experiencing avoidable side effects and incurring additional expenses. Similarly, sentinel node biopsy has low sensitivity for predicting mortality in individuals over 60 years old. Therefore, regarding the two groups, management can be achieved without SLN biopsy via wide local excision [90].

Several prediction models for nodal invasion have been proposed to ensure the safe performance of SLN biopsy and regional lymphadenectomy, considering potential complications such as hemorrhage, infection, dehiscence, lymphocele, lymphedema, and neuropathies [89,91]. The CP-GEP model incorporates age, Breslow thickness, and the expression levels of eight genes (GDF15, CXCL8, LOXL4, TGFBR1, ITGB3, PLAT, SERPINE2, and MLANA). This model fulfills its role both by reducing the number of SLN-negative biopsy procedures and by identifying with increased accuracy patients with even a low risk of nodal metastasis [91,92].

## 3. Molecular Signaling and Tumor Microclimate

The most common pathogenic pathway in the development of cutaneous melanomas (90% of cases) is that of the mitogen-activated protein kinase (MAPK) cascade. The activation process occurs when growth factors are connected to tyrosine kinase receptors, leading to the activation of G protein monomers, which are part of the RAS family. In turn, they activate the serine/threonine kinase cascade that activates ERK. ERK triggers the activation of transcription factors, which in turn leads to cell growth, proliferation, and migration [93,94]. In addition to the previously mentioned effects, cell cycle disturbances and the inhibition of apoptosis are added [92].

BRAF gene mutations are noted in 60% of melanomas. They are most frequently associated with the young population and with intermittent exposure to the sun. The BRAF gene has the role of encoding serine/threonine kinases, being involved in the MAPK pathway. Around 80% of BRAF mutations occur due to a substitution of glutamic acid with valine (known as V600E). This change activates ERK and MAPK, leading to a series of cellular responses. Valine can be substituted to lysine (V600K) in 20% of cases or to arginine (V600R) in 7% of cases. It should be emphasized that nevi also have the BRAF V600E mutation, resulting in the fact that only this mutation does not lead to melanogenesis [95,96]. It should be mentioned that melanomas with BRAF V600K mutation present several peculiarities. These have an increased prevalence in the elderly and those with chronic solar skin damage. In contrast to other mutations, there is a noticeably higher frequency of localization in the head and neck, and a significantly shorter disease-free interval until the first metastasis [97].

In 20% of cases, the NRAS gene mutation is noted. The most frequent mutations occur at codon 61, and less often at codon 12. Mutations are responsible for activating both the MAPK pathway and the PI3K-AKT pathway [98]. Elderly individuals who have chronic exposure to the sun are more likely to develop aggressive melanomas with NRAS mutations, leading to an increased rate of progression due to higher proliferative activity [99]. NRAS mutations, just like BRAF V600E mutations, do not solely support oncogenesis; they also occur in benign lesions known as congenital nevi [95]. To inactivate the domain, it is necessary to convert RAS-GTP to RAS-GDP. Mediation is carried out through the NF1 gene. Mutations in NF1 are present in up to 15% of melanomas, and their loss of function results in the activation of the MAPK and PI3K pathways [98,100].

NRAS or NF1 mutations are responsible for the activation of the PI3K pathway. After this sequence of events, PIP2 is phosphorylated to PIP3, enabling it to bind to a domain of AKT. The combined activation of AKT and the inhibitory stimulus of the tuberous sclerosis complex (TSC1/TSC2) results in the activation of the mTOR complex [98,101]. MTORC1 is the key element within the complex, driving the production of biomass and energy essential for powering cell growth and proliferation. Additionally, it is crucial not to overlook the impact of mTORC2 on inhibiting apoptosis, promoting cell migration, and reorganizing the cytoskeleton [102,103]. In conclusion, the activation of the AKT/mTOR axis is accountable for the aggressiveness of melanoma due to cellular nutritional support and its distant metastasis [104].

Another way to activate the PI3K pathway is represented by the inactivation of PTEN. PTEN acts as a catalyst for the conversion of PIP3 to PIP2 by blocking the pathway [98]. Chronic exposure to UV radiation induces a decrease in PTEN expression. In addition to this aspect, mutations leading to the inactivation of the PTEN gene have also been identified. Consequently, the loss of PTEN leads to the activation of the PI3K/AKT/mTOR pathway [105]. The absence of the PTEN protein has a significant impact on the tumor microenvironment by reducing the presence of T lymphocytes, including cytotoxic T cells [106].

Up to 78% of melanomas have the TERT promoter mutation. It is often observed in skin lesions resulting from prolonged sun exposure, serving as a clear indication of UV radiation, but it can also appear in individuals with no history of chronic exposure. Normally, telomerase activity is inhibited. It is active in cells with continuous division (hematopoietic cells, stem cells, and germ cells). Cells with promoter mutations possess telomeric elongation, responsible for prolonged survival and abnormal multiplication capacity. TERT promoter mutations are associated with BRAF and NRAS mutations. The upregulation of telomerase expression enhances the stability of the altered genome after the mutations of the two genes mentioned earlier [107,108].

KIT is a transmembrane receptor tyrosine kinase present in melanocytes. Mutations in cutaneous melanomas are predominantly observed in acral melanomas, as well as those that arise from chronic UV exposure [109]. The activation of c-KIT leads to the recruitment of proteins containing the SH-2 domain. These components will trigger the RAS domain, leading to the activation of BRAF and entry into the MAPK pathway. These proteins can all bind to the p85 subunit of the PI3K pathway, leading to the activation of the AKT/mTOR complex [110].

The activation of KIT results in the amplification of MITF [98]. MITF is a transcription factor responsible for melanocyte differentiation [111]. This gene, known as the master gene of melanocyte homeostasis, plays a crucial role in regulating the function, survival, and proliferation of the melanocyte [112]. During pathogenesis it appears in the advanced stages, occurring in 15–20% of melanomas, and especially in metastatic ones [111]. Its expression has three distinct phenotypes: it is reduced during the invasion phase, moderate during the proliferative phase, and significantly increased during the differentiation phase [113]. MITF is also involved in the MAPK pathway. It can act as an antiproliferative transcription factor by terminating the cell cycle. However, in the case of BRAF mutations, this does not occur. On the contrary, an increased number of proteins responsible for cell survival and proliferation will be formed and maintained [111]. Increased levels of MITF expression affect the tumor microclimate, decreasing immune cell infiltrate. This effect is reflected in the low survival of patients [114].

RAC1, a member of the small GTPases family, plays a crucial role as an effector in various essential processes such as proliferation, survival, inflammatory response, and differentiation [115]. Its mutations are found in approximately 5% of melanomas, especially those with sun exposure. In the oncogenetic process, its action results in a more accelerated conversion of GDP with GTP. This characteristic means that it is involved in the addition of mutations to the BRAF or RAS genes, and/or in deletions of the NF1 or PTEN genes, indirectly involving the MAPK and PI3K pathways [98,115].

Another tyrosine kinase receptor involved in melanogenesis is represented by c-MET. Its activation is mainly due to the excessive oversecretion of HGF (its ligand). It is produced by the cells present in the tumor microclimate [111]. The heightened activity of HGF/c-MET drives the proliferation of melanocytes, enhances their invasive potential, and provides protection against apoptosis. Their overexpression also activates the MAPK and PI3K/AKT pathways [116].

Until now, only certain genes and pathogenic pathways have been targeted in the treatment of cutaneous melanoma. These include the BRAF gene, the MEK/MAPK pathway, the NRAS gene, and the KIT gene (Table 3).

The immune system plays a crucial role in the development of melanoma, alongside genetic changes. So-called anti-melanoma immunity responds through humoral and cell-mediated immunity. The cancer cell has to adapt to survive. These adaptation mechanisms include reduced antigen presentation, impaired T lymphocyte function, compromised immunological barriers in the tumor microenvironment, and disruption of T-cell-pathway regulatory mechanisms [73]. A balance between pro- and anti-inflammatory activity is achieved by the immune checkpoint. The immune checkpoint consists of pathways that can either inhibit or stimulate the activity of immune cells [117]. Recent research has emphasized the critical role of two proteins, CTLA-4 and PD-1, in cancer immunotherapy [118]. CTLA-4 functions by delivering an inhibitory signal that terminates the immune response, thus negatively modulating the activity of T lymphocytes [119]. For T lymphocytes to become activated, both the TCR and the transmembrane protein CD28 need to receive two signals from the ligands B7.1 (CD80) and B7.2 (CD86). CTLA-4 shares a similar structure with CD28 and interacts with B7 family ligands, but it has a higher affinity. However, its mode of action is the opposite. CTLA-4 inhibits TCR action and stimulates AKT action, resulting in an immortal cell with sufficient energy [119,120,121].

PD-1 is a protein belonging to the immunoglobulin superfamily that is found in activated T lymphocytes, B lymphocytes, natural killer lymphocytes, dendritic cells, and macrophages [118]. This protein presents two ligands: PD-L1 (CD274) and PD-L2 (CD273). T cells, B cells, and macrophages express PD-L1, while activated macrophages and dendritic cells express PD-L2 [122]. At the T-cell level, the PD-1/PD-L1 complex plays a critical role in transmitting antiapoptotic signals to tumor cells. This process limits cytokine production and promotes heightened resistance against killer T cells, known as adaptive resistance [123,124,125]. Within the tumor microclimate, upregulated PD-L1 prevents inflammation to limit tissue damage. Another important role in this is providing negative feedback to suppress tumor immunity [124]. A separate event is the interaction with CD80, together helping to inhibit the activity of T cells. The role of PD-L2 is a controversial one, its function being both stimulatory and inhibitory to helper T cells [123].

## 4. Treatment of Cutaneous Melanoma

The first-line treatment in early melanomas (stages 0-IIA) is surgical. The National Comprehensive Cancer Network suggests different excision margins based on the thickness of the melanoma: 0.5 cm for melanoma in situ, 1 cm for melanomas up to 2 mm thick, and 2 cm for thicknesses over 2 mm [126,127]. In the case of invasive melanomas, it is recommended to perform wide local excision, which should include excision of the subcutaneous tissue up to the level of the fascia to ensure thorough removal. Of course, if invasion of the fascia is observed, it and the underlying tissue will be excised. In acral melanomas, depending on their invasiveness, amputations can be reached [127].

Surgical excision can be performed in almost all cases only with local anesthesia. If the surgical margins are not free of the tumor, re-excision is required. Extended surgical margins are not recommended in the case of free histological margins. Surgical excision is generally not performed under two circumstances: when the patient explicitly declines the procedure or when the patient’s overall health is severely compromised [128]. Complete lymph node dissection has the role of preventing the expansion of tumor cells and increasing the accuracy of the melanoma stage diagnosis. However, the German Dermatologic Cooperative Oncology Group Selective Lymphadenectomy (DeCOG), Multicenter Selective Lymphadenectomy Trial (MSLT-2), and other studies, did not observe a benefit in terms of overall survival or melanoma-specific survival of patients. Moreover, the rate of complications was higher compared to the cases biopsied for SLN or the observational groups [128,129].

Adjuvant therapy is essential for advanced melanomas, particularly those in stages IIB to IV and those with a thickness beyond 2 mm. This therapy offers a range of treatments including interferon, IL-2, targeted therapy, and/or immunotherapy [126]. A special mention should be made regarding stage III treatment with a combination of immune checkpoint inhibitors (anti-PD-1) and targeted therapy (BRAF + MEK inhibitor). In the study conducted by Helgadottir H et al., adjuvant therapy highlighted a net benefit in terms of recurrence-free survival. Apparently, in terms of overall survival, the results did not have the expected positive results [130].

Interferon has proven its effectiveness through its multiple mechanisms. It possesses an immunomodulatory effect, augmenting the expression of class I of the histocompatibility complex, inhibiting proliferation, triggering apoptosis, and diminishing VEGF secretion. Furthermore, its impact extends to immune cells, as it stimulates the conversion of helper 2 lymphocytes into Th1 cells, suppresses regulatory T lymphocytes, enhances the cytotoxic activity of T lymphocytes, boosts the survival rate of dendritic cells, and amplifies the cytotoxic activity of natural killer cells [131]. These effects result in medication with interferon α-2b reducing the risk of recurrence, but have minimal benefits for survival [132]. The pegylated form (PEG-IFN) offers enhancements such as an optimized pharmacokinetic profile, prolonged half-life, and weekly administration [133]. Regrettably, there is no disparity in terms of overall patient survival between the two forms [132]. Clinical trials were performed to highlight the difference between low doses and high doses of IFN α-2b. The low doses did not bring obvious improvements, and the increased ones caused increased toxicity and numerous adverse reactions. Thus, interferon therapies have a moderate efficiency, not reflecting a major effect [134].

Treatment with cytokines (IL-2) stimulates the production of lymphokine-activated killer cells. Also, IL-2 potentiates the growth factors of T cells and the cytolytic effect of natural killer cells [135,136]. Optimal treatment necessitates higher doses and, by extension, close monitoring of the patient. This is due to the many adverse reactions it possesses. The most common are represented by fever, hypotension, oliguria, dyspnea, tachypnea, neurotoxicity, pruritus, neutropenia, and thrombocytopenia. However, the advantages of this treatment include an impressive response rate of 15–20%, a notable complete response rate of 5–10%, and a remarkably favorable survival rate. Monotherapy treatment is becoming less common these days. It is now more common to either replace it with immunological therapy or use it in combination with this [137].

Treatment with alkylating agents has been a common method directed against advanced melanoma since the 1970s. The most used chemotherapeutic agent is Dacarbazine. This is a prodrug whose activation is initiated by the liver. The objective response rate is up to 15%; most responses are partial, and only approximately 3–5% are complete. It is relatively well tolerated, with its toxicity being grade 3 or 4 in about 18% of patients. The most frequent adverse effects include nausea, vomiting, fatigue, and myelosuppression [138,139]. Other chemotherapies used are represented by temozolamide and fotemustine. These are chosen especially in cases of cerebral metastasis [138,140].

A therapeutic advance in contrast to chemotherapy is the use of targeted therapy. It has greater specificity and different side effects compared to alkylating agents [141]. Targeted therapy involves using specific substances to target and block the growth and spread of cancer cells by focusing on certain molecules. This concept is based on good knowledge of the physiopathogenesis of the tumor for which it is used. Therapy can act on cancer cells and/or on the tumor microclimate. Within these therapies, you can use small molecules, therapeutic monoclonal antibodies, gene therapy, or therapeutic cancer vaccines [142]. In the treatment of melanoma, the most used small molecules are directed against the BRAF gene (Vemurafenib, Dabrafenib, and Encorafenib) and/or MEK/MAPK (Binimetinib, Cobimetinib, Trametinib) [143].

Vemurafenib is a therapeutic agent directed against melanoma with BRAF V600E mutation in advanced stages. Clinical studies have observed a 63% reduction in the risk of death and a 74% reduction in tumor progression. Adverse effects are manageable and directly proportional to dose and exposure. The skin, liver, central nervous system, and joints are the areas most commonly impacted. Squamous cell carcinoma poses the most significant risk, likely due to a paradoxical activation of the MAPK pathway [144,145].

Dabrafenib is also directed against advanced-stage melanoma with BRAF mutation (V600E and V600K). In cases without brain metastases, the response rate is 50%. Progression-free survival and overall survival demonstrate a stable phase lasting 3 years, with a progression-free rate of 11–12% at 5 years. In the case of cerebral metastases, regardless of the local treatment performed or not performed, the efficiency decreases. The median survival for the V600E mutation is higher than for the V600K mutation, with 7.2–7.6 months compared to 3.7–5 months, respectively. In addition, the frequency of adverse reactions is also higher in the case of the presence of brain metastases. Common adverse reactions to the treatment include headache, hyperkeratosis, fever, arthralgia, hair loss, fatigue, and hyperkalemia [146,147].

Encorafenib is the most recently approved targeted agent against melanomas in advanced stages with BRAF mutation. Most treatment schemes associate it with the administration of an MEK inhibitor. Clinical studies have demonstrated an impressive 60% objective response rate, as well as a progression-free survival rate that outperforms the other two BRAF inhibitors. Grade 3 or 4 adverse effects are rare. Nausea, diarrhea, vomiting, fatigue, joint pain, and headache are among the most frequent reactions [148,149].

Binimetinib is a selective non-competitive ATP inhibitor of MEK1 and MEK2. It is often associated with Encorafenib, bringing improvements to both progression-free survival and overall survival. Its main adverse effects are rash, diarrhea, nausea, acneiform dermatitis, and fatigue [150,151].

Cobimetinib is an MEK1 and MEK2 inhibitor, allosteric, reversible, and non-competitive ATP. It is administered together with Vemurafenib. The combination of the two results in maximum efficiency, increasing apoptosis and inhibiting tumor growth. The majority of adverse reactions are mild to moderate, including symptoms such as diarrhea, nausea, vomiting, skin rash, arthralgia, and increased levels of creatine kinase. In the phase 3 clinical trials, an objective response rate of 68–87% was accomplished, with a remarkable 10% of participants showcasing a complete response. Notably, the average progression-free survival ranged from 9.9 to 13.7 months [152,153].

Trametinib is a non-competitive ATP-selective MEK1/2 inhibitor, approved both as a single agent and in combination with Dabrafenib. It decreases tumor proliferation by arresting the cell in G1 of the cell cycle, causing apoptosis. Treatment may need to be interrupted or delayed due to adverse effects such as cardiomyopathy, retinal pigment epithelial detachment, retinal vein occlusion, and febrile reaction. The association with Dabrafenib brings both antitumor benefits and the improvement of patients’ quality of life. This association delivers a response rate of 64–67%, along with an average progression-free survival of 9.3 to 11.4 months [154,155,156].

Regarding other elements of the pathogenic chain, clinical studies’ outcomes have not yielded the anticipated results for the targeted treatment. Despite being one of the first genes discovered in the development of melanoma, there has been no advancement in the development of an NRAS inhibitor until now. Drugs such as Binimetinib or farnesyltransferase inhibitors can be used on RAS mutations [136,157,158]. Due to the complex downstream protein shifting process, developing a drug against RAC1 is an extremely challenging task [136]. Medications against the KIT mutation exist on a large scale (Imatinib, Sunitinib, Dasatinib, Nilotinib), but in the case of melanomas, they have not proven their effectiveness [136,159,160].

Currently, immune checkpoint inhibitors are among the most used anti-melanoma medications. They can be administered as a monotherapy or in combination with targeted therapies. Their advantage lies in the ability to induce lasting control over the pathology [141,161]. The National Comprehensive Cancer Network recommends Ipilimumab, Nivolumab, and Pembrolizumab as adjuvant treatments for advanced melanomas [162].

Ipilimumab is a CTLA-4 inhibitory monoclonal antibody. It can be used alone or in combination with Dacarbazine (it has better survival rates alone than with Dacarbazine, but results in more frequent grade 3 and 4 adverse effects) or with Nivolumab (which presents synergistic effects). Monotherapy has a complete response rate of 6%, with an average survival of 19.9 months and a 5-year survival of 26%. In association with Nivolumab, the complete response rate rises to 22%, the average survival exceeds 60 months, and the 5-year survival is 52% [161,163].

Nivolumab was the second PD-1 inhibitory monoclonal antibody used in advanced melanomas. It is usually used as a second-line treatment, after anti-CTLA-4 or anti-CTLA-4 and BRAF inhibitor treatment [164]. The complete response rate is 19%, with an average survival of 36.9 months and a 5-year survival of 44% [161].

Pembrolizumab is a PD-1 inhibitory IgG4 monoclonal antibody. It has the role of potentiating the production of IL2, IL6, IL17, ɤ-interferon, and α-tumor necrosis factor. The most common adverse reactions are represented by pruritus, rash, diarrhea, arthralgias, and nausea. A disadvantage of using Pembrolizumab consists of immune-type adverse reactions (hypo- or hyperthyroidism and pneumonitis) [165]. A prediction score of immune events is CYTOX. It is composed of 11 circulating cytokines: IL-1a, IL-1B, IL-1RA, IL-2, IL-12p70, IL-13, IFN-α2, FGF-2, Fractalkine, G-CSF, and GM-CSF. Their increase has been associated with a severe risk of developing immune-type adverse reactions and requires the administration of immunosuppressive agents [166]. Unlike CTLA-4 inhibitors, this medication shows increases in progression-free survival and total survival [167]. Patients treated with Pembrolizumab have an average survival of 38.7 months, with a 5-year survival rate of 43%. An advantage also lies in the increased response rate (22%) in cases of brain metastases [161].

In the case of immune therapies, preclinical studies have shown that preoperative administration leads to increased survival. There are hypotheses suggesting that even a single dose of a PD-1 inhibitor could potentially stimulate the activity of cytotoxic lymphocytes [168]. Moreover, in the case of Pembrolizumab, randomized clinical trials have observed an increased efficiency of neoadjuvant administration. This involves activating lymphocytic infiltration in the tumor, exposing the antigen, and reducing the tumor size during surgery [163].

Currently, 20 clinical trials in phases 2 and 3 are ongoing and active with disseminated results, and are monitoring the effectiveness (objective response rate, survival rate, relapse, progression-free survival) of various therapies against cutaneous melanomas (Table 4). Studies that aimed for response rate or clinical benefit over a time period are reported as percentages, and those that aimed for overall survival, progression-free survival, or recurrence-free survival are noted as time periods [169].

The factors that influence the response to treatment with immune checkpoint inhibitors are varied. The index that quantifies all tumor mutations is called tumor mutational burden. A correlation of this index with the response rate was observed in anti-PD-1 and anti-CTLA-4 therapies [170]. Another element that influences the treatment is represented by the major histocompatibility complex (MHC). MHC I controls the function of CD8 lymphocytes, which directly target cancer cells, while MHC II facilitates the activity of CD4 lymphocytes, which promote an inflammatory response by producing ɤ-interferon [171,172]. MHC I activity serves as a predictor for anti-CTLA-4 therapy, while MHC II predicts the efficacy of anti-PD-1 therapy [170]. Nevertheless, the absence of the B2M protein from MHC I results in a resistance mechanism against both types of inhibitors [173]. Another predictor of response to anti-PD-1 therapy is represented by the immunohistochemical expression of its ligand (PD-L1). Positivity is determined by a threshold of either greater than 1% or greater than 5% of tumor cells [161,170]. However, currently, no study has identified differences in the overall survival of patients depending on PD-L1 expression. Therefore, quantifying the expression is optional and should not be considered when making therapeutic decisions for stage IV cases [174]. The gastrointestinal microbiota play an important role in the response to immune checkpoint inhibitors. The presence of a population rich in *Faeclibacterium* spp. shows a favorable clinical response, while the abundance of *Bacteroides* spp. is associated with a low response to ICI [170].

Two of the most prevalent factors in resistance to treatment with inhibitors targeting the PD-1/PD-L1 axis involve the depletion of T cells and the impaired function of tumor-infiltrating lymphocytes. The depletion of T cells in the tumor microenvironment is caused by damage to the immunoreceptor tyrosine-based inhibitory motif domain. The consequences consist of the impairment of PD-1 signaling and the activity of T lymphocytes. The overexpression of TIM-3 in regulatory T lymphocytes causes the dysfunction of tumor-infiltrative lymphocytes, leading to resistance to treatment [175,176]. Cells refractory to treatment have undergone mutations, losing their response to ɤ-interferon or MHC class I [177].

A series of investigations can be used to monitor the effectiveness of the treatment. The serum LDH test is the most easily accessible. Increased levels can serve as both a reliable indicator of recurrence, with a sensitivity of 72% and a specificity of 97%, and a surrogate for a high index of tumor mutational burden [166,170]. Increased basal levels of IL-6 were associated with a low therapeutic response and low patient survival [170]. Circulating tumor DNA detected in the patient’s serum correlates directly proportionally with progression and tumor mutational burden. An additional indication of improved treatment adherence is reflected in the heightened presence of CD8-positive lymphocytes within the tumor microenvironment and the rise in T helper 9 lymphocytes in the bloodstream [177].

There are several other noteworthy treatments, such as T-VEC, ECT, and Treg inhibitors [178,179]. T-VEC is an oncolytic viral therapy that can be used in grade IIIB-IV melanomas. Because its effectiveness is not high, it is only used in specific subgroups of patients who have only local or regional cancer extension [178]. In phase III of the MASTERKEY-265 clinical trial, the combination of T-VEC and Pembrolizumab did not result in significant improvements in either progression-free survival or overall survival compared to the combination of Pembrolizumab and the placebo. Even though progression-free survival was higher by 5.8 months in T-VEC and Pembrolizumab, this was not reflected in overall survival [180]. ECT is a technique that uses high-intensity electrical pulses intending to deliver medication (cytotoxic, cisplatin, and bleomycin) to tumor cells. Following the action, the lymphatic vessels are destroyed, and the local recurrence rate is zero [179]. Treg inhibitors have the role of stimulating antitumor immunity. This treatment targets the tumor microenvironment to prevent the infiltration of regulatory T lymphocytes into the tumor tissue [181]. A particular aspect is represented by adoptive T-cell therapy. An increased number of lymphocytes are selected from the tumoral lymphocytic infiltrate. They are cultivated in vitro, and are capable of recognizing and performing antitumor functions. After that, they are re-administered [135].

Also, new therapeutic approaches regarding advanced melanomas are underway: anti-LAG3, GITR agonists, and anti-TIGIT. LAG-3 (lymphocyte activation gene 3) is an inhibitory receptor of the immune checkpoint of CD4+/CD8+ and Treg T lymphocytes. It suppresses the activation and proliferation of T lymphocytes. The coexistence of LAG-3 and PD-1 leads to the persistent stimulation and subsequent exhaustion of T lymphocytes, which may present a possible mechanism of resistance to immunotherapy [182,183]. GITR (the glucocorticoid-induced TNF receptor) is a member of the TNF receptor superfamily. Activating the GITR pathway stimulates antitumor activity by promoting proliferation and enhancing the effector functions of CD4+ and CD8+ T lymphocytes. Also, the effects downregulate the immunosuppressive activity of Treg lymphocytes [182]. TIGIT (T cell immunoreceptor with immunoglobulin and ITIM domain) binds to CD112 and CD155 ligands that downregulate the functions of T lymphocytes and natural killer cells. The inhibition of PD-1 and TIGIT potentiates the activity of tumor antigen-specific CD8+ T lymphocytes and tumor-infiltrating lymphocytes [182,184].

The study’s limitations arise from the narrative nature of the review, which can potentially introduce bias due to the absence of specific search criteria and the inclusion of less relevant articles in the field. However, we assume that the entire study was based on both the most recent bibliographic sources and the most cited works so that we have a citation pool as current as possible and with increased relevance. Furthermore, we strive to maintain objectivity by preserving the true essence of the findings from the referenced studies, refraining from introducing our interpretations or perspectives. By this, we tried not to change the interpretations of the original studies. Despite the lengthy nature of the study, we believe that the careful selection and consolidation of all available information truly enhances the reader’s understanding of the chronological sequence of events that gives rise to skin melanomas. Moreover, the current study effectively synthesizes the key existing therapeutic approaches, outlining their advantages and disadvantages, while also shedding light on potential treatments currently under investigation in clinical trials.

## 5. Conclusions

Cutaneous melanoma remains a cancer with a consistently rising occurrence. Despite the progress made, the presented data still form an incomplete puzzle. Many of the risk factors cannot be avoided, despite all the efforts made for prevention. The clinical elements are the ones that attract attention, but unfortunately they are ignored in most cases. Histopathological aspects form the final diagnosis. The initial microscopic criteria are crucial in revealing the hidden, bleak image concealed within a pigmentary lesion, which may sometimes seem insignificant at first glance. The pathogenic ways of development are varied and, even if they are known, they still require further study. Despite therapeutic advances (targeted therapy and immune checkpoint inhibitors), the mortality rate is still high. Many patients cannot benefit from the latest generation of therapies. Currently, numerous clinical studies are being conducted to improve therapeutic weapons. However, there is still a need for thorough research on evolutionary mechanisms for the development of new drugs. Developing new therapeutic solutions, such as highly efficient drugs or advanced nanoparticles, that can enhance patient outcomes should be a top priority. These innovations should not only minimize adverse effects, but also improve survival rates and prognosis.

## Figures and Tables

**Table 1:** Summary of cutaneous melanoma detection algorithms.

Algorithm	Characteristics	Sensitivity Specificity
**ABCD(E)**	Assess: asymmetry (A), borders (B), color (C), diameter (D), elevation or evolution (E).	82.6–92.8% 70–91.2%
**Glasgow**	Major criteria: shape, color, change in size; Minor criteria: diameter ≥ 7 mm, sensory change, crusting or bleeding, the presence of signs of inflammation.	NA *
**Menzies**	Melanoma is suspected if there are 2 out of 2 negative aspects and at least 1 positive aspect: Negative aspects: symmetry and the presence of a single color; Positive aspects: depigmentation, black dots or globules, multiple colors, multiple blue-grey dots, or a broadened network.	85.7 71.1%
**CASH**	A score of ≥8 points is suggestive of melanoma: Color (1 point for each): blue, white, red, black, dark brown, light brown; Homogeneity (1 point for each): polymorphous blood vessels, blotches, regression structures, blue-white veil, pseudopods, globules, atypical network; Symmetry: biaxial asymmetry (2 points), monoaxial symmetry (1 (point), and biaxial symmetry (0 points); Architectural disorganization: marked (2 points), moderate (1 point), and none/mild (0 points).	68% 98%
**Seven-point Scale**	A score of ≥3 points is suggestive of melanoma: Major criteria (2 points): atypical pigmentation, atypical pattern, and blue-white veil; Minor criteria (1 point): irregular streaks, irregular pigmentation, irregular globules or spots, and areas of regression.	83.6% 71.5%
**Three-point Checklist**	Asymmetry in color and/or structure in one or two axes; Any blue and/or white structure in the lesion; A pigmented network with thickened lines and irregular distribution	91% 71.9%

* NA—Not available.

**Table 2:** Clinical–pathogenic correlations in non-familial cutaneous melanomas [66,70,71,72,73,74].

Subtype of Melanoma	UV Exposure	Precursor Lesion	Genes Involved
Superficial spreading melanoma	Low	Nevus or none (de novo)	BRAF V600E, or NRAS, TERT
Nodular melanoma	Low or high	Pigmented nevus or none (de novo)	BRAF V600E, NRAS, TERT, KIT, NF1
Lentigo maligna melanoma	High	Lentigo maligna	NF1, NRAS, BRAF, KIT, TERT, RAC1
Acral melanoma	No exposure	Acral nevus	NRAS, KIT, NF1, BRAF, TERT

**Table 3:** Drugs are available depending on the gene and pathogenic pathway involved in the development of cutaneous melanoma.

Gene/Pathogenic Pathway	Available Drugs
BRAF V600E	Vemurafenib, Dabrafenib, Encorafenib
BRAF V600K	Dabrafenib
MEK/MAPK	Binimetinib, Cobimetinib, Trametinib
NRAS	Binimetinib, Farnesyltransferase inhibitors
KIT	Imatinib, Sunitinib, Dasatinib, Nilotinib

**Table 4:** Active clinical trials with published results evaluating treatments for cutaneous melanoma [169].

NCT Identifiers	Phase	Estimated Study Completion	Condition/Disease	Primary Objective (Time Frame)	Study Arms (Patients)	Results
NCT04068181	2	26 February 2024	Stage III B-IV M1d for whom surgery is not recommended	Objective response rate (17.48 months)	Talimogene laherparepvec + Pembrolizumab Locally recurrent/metastatic − primary resistance (26)	0%
					Talimogene laherparepvec + Pembrolizumab Locally recurrent/metastatic -acquired resistance (15)	6.7%
					Talimogene laherparepvec + Pembrolizumab Adjuvant setting—disease-free interval < 6 months (15)	40%
					Talimogene laherparepvec + Pembrolizumab Adjuvant setting—disease-free interval ≥ 6 months (15)	46.7%
NCT03698019	2	30 April 2024	Acral lentiginous melanoma, stage III–IV	Event-free survival rate (2 years)	Adjuvant Pembrolizumab, 3 weeks for 18 cycles (159)	49%
					Adjuvant Pembrolizumab, 1 every 3 weeks for 3 cycles, and neoadjuvant Pembrolizumab 3 weeks for 15 cycles (154)	72%
NCT00539591	2	May 2026	Stage IIC, III, IV or recurrent cutaneous melanoma, up to 21 years	Tumor response rate (8 weeks)	Temozolomide/Peginterferon a-2b with measurable disease (2)	0 participants
NCT02743819	2	June 2026	Cutaneous melanoma with disease progression or stable disease	Overall response rate (16 weeks)	Pembrolizumab + Ipilimumab (70)	20 (28.6%) participants
NCT03149029	2	31 December 2024	Metastatic or unresectable cutaneous melanoma	The rate of clinical benefit (6 months)	BRAFV600 Mutant: Pembrolizumab + Dabrafenib + Trametinib (14)	5 (35.7%) patients
					BRAFV600 Wild Type: Pembrolizumab + Trametinib (0)	0 patients
NCT02581930	2	Not Provided	Stage IV disease, disease refractory (cutaneous melanoma)	Estimate rate of objective response (1 year)	Ibrutinib (18)	0 (0%) participants
NCT00937937	2	Not Provided	Acral lentiginous melanoma, cutaneous nodular melanoma, lentigo maligna melanoma, low-CSD melanoma, stage IV disease	Overall survival (up to 3 years)	Dinaciclib iv * (72)	8 months
NCT01134614	2	Not Provided	Metastatic cutaneous melanoma, recurrent cutaneous melanoma, unresectable cutaneous melanoma (stage III, IIIA, IIIB, IIIC, IV)	Overall survival (5 years)	Ipilimumab + Sargramostim (123)	17.5 months
					Ipilimumab (122)	12.7 months
NCT01708941	2	Not Provided	Stage III or stage IV cutaneous melanoma, either initial presentation or recurrent	Progression-free survival (up to 10 years)	Ipilimumab + Recombinant Interferon alfa-2b (37)	7.5 months
					Ipilimumab (44)	4.4 months
NCT02967692	3	29 March 2024	Unresectable or metastatic cutaneous melanoma with BRAF V600 mutation	Progression-free survival (2.8 years)	Spartalizumab + Dabrafenib + Trametinib (267)	16.2 months
					Placebo + Dabrafenib + Trametinib (265)	12.0 months
NCT02908672	3	31 March 2024	Stage IV (metastatic) or unresectable stage III C (locally advanced) cutaneous melanoma	Progression-free survival (33 months)	Atezolizumab + Cobimetinib + Vemurafenib + Vemurafenib Placebo (256)	15.1 months
					Atezolizumab Placebo + Cobimetinib + Vemurafenib (258)	10.6 months
NCT02388906	3	6 October 2024	Stage IIIb/C or stage IV before complete resection of cutaneous melanoma	Recurrence-free survival (up to 36 months)	Nivolumab 3 mg/kg (452)	52.37 months
					Ipilimumab 10 mg/kg (453)	24.08 months
NCT01844505	3	31 October 2024	Stage III (unresectable) or stage IV cutaneous melanoma	Progression-free survival (20 months)	Nivolumab monotherapy once every 2 weeks (316)	6.87 months
					Nivolumab + Ipilimumab once every 3 weeks for 4 doses followed by Nivolumab (314)	11.50 months
					Ipilimumab monotherapy, 3 mg/kg, iv *, for a total of 4 doses (315)	2.89 months
NCT00003641	3	October 2025	Melanoma of cutaneous origin (stage II, III, IV)	5-year relapse-free survival rate (5 years)	Observation (569)	0.7 proportion of participants
					High-dose interferon alfa-2b, iv * (581)	0.7 proportion of participants
NCT03470922	3	16 December 2025	Stage III (unresectable) or stage IV cutaneous melanoma	Progression-free survival (33 months)	Relatlimab + Nivolumab, 1:3 ratio, every 4 weeks (355)	10.12 months
					Nivolumab 4 weeks 359	4.63 months
NCT02362594	3	31 July 2026	Completely resected stage III cutaneous melanoma	Recurrence-free survival (6 months)	Pembrolizumab 200 mg, iv (264)	82.2% participants
					Placebo iv * (280)	73.3% participants
NCT04099251	3	29 June 2027	Resected, stage IIB/C cutaneous melanoma with negative sentinel lymph node biopsy	Recurrence-free survival (32 months)	Nivolumab iv *, 4 weeks for 12 months (526)	28.52 months to NA **
					Placebo (264)	21.62 months to NA **
NCT03553836	3	12 October 2033	Stage IIB or IIC cutaneous melanoma	Recurrence-free survival (up to ~32.7 months)	Pembrolizumab, every 3 weeks for up to 17 cycles (487)	NA **
					Placebo (489)	NA **
NCT01274338	3	Not Provided	Recurrent cutaneous melanoma (stage IIIB, IIIC, IV)	Recurrence-free survival (up to 8 years)	High-dose recombinant Interferon alpha-2b (528)	2.5 years
					Low-dose Ipilimumab (523)	4.5 years

* iv—intravenous; ** NA—not available (due to insufficient number of events).

## Data Availability

Dataset available on request from the authors.

## Abbreviations

ACD	Adrenocortical dysplasia protein homolog
AKT	Serine–threonine protein kinase
ATP	Adenosine triphosphate
BAP1	Breast cancer 1 gene-associated protein 1
BRAF	v-raf murine sarcoma viral oncogene homolog B1
BRCA2	Breast cancer 2 gene
CD	Cluster of differentiation
CDK4	Cyclin-dependent kinase 4
CDKN2A	Cyclin-dependent kinase inhibitor 2A
c-MET	Receptor tyrosine kinase
CP-GEP	Clinicopathologic and gene expression
CPD	Cyclobutane pyrimidine dimer
CTLA-4	Cytotoxic T-lymphocyte-associated protein 4
CXCL8	C-X-C motif chemokine ligand 8
DNA	Deoxyribonucleic acid
DeCOG	German Dermatologic Cooperative Oncology Group Selective Lymphadenectomy
ECT	Electrochemotherapy
ERK	Extracellular signal-regulated kinase
FGF	Fibroblast growth factors
G-CSF	Granulocyte colony-stimulating factor
GDF15	Growth/differentiation factor 15
GDP	Guanosine diphosphate
GITR	The glucocorticoid-induced TNF receptor
GM-CSF	Granulocyte–macrophage colony-stimulating factor
GTP	Guanosine triphosphate
HGF	Hepatocyte growth factor
HIV	Human immunodeficiency virus
IFN	Interferon
IL	Interleukin
ITGB3	Integrin subunit beta 3
iv	Intravenous
ITA	Individual typology angle
KIT	Proto-oncogene c-KIT
LAG-3	Lymphocyte activation gene 3
LDH	Lactate dehydrogenase
LOXL4	Lysyl oxidase-like 4
MAPK	Mitogen-activated protein kinase (MEK)
MHC	Major histocompatibility complex
MITF	Melanocyte-inducing transcription factor
MLANA	Melan-A
MSLT-2	Multicenter Selective Lymphadenectomy Trial
mTOR	Mammalian target of rapamycin
mTORC	Mammalian target of rapamycin complex
NA	Not available
NADPH	Nicotinamide adenine dinucleotide phosphate
NF1	Neurofibromatosis type 1
PD-1	Programmed cell death protein 1
PD-L	Programmed death-1 ligand
PI3K	Phosphoinositide 3-kinase
PIP2	Phosphatidylinositol 4,5-bisphosphate
PIP3	Phosphatidylinositol (3,4,5)-trisphosphate
PLAT	Plasminogen activator, tissue type
POT1	Protection of telomeres 1
PTEN	Phosphatase and tensin homolog
PUVA	Psolaren and ultraviolet A
RAC1	Rac family small GTPase 1
RAS	Rat sarcoma virus
SERPINE2	Serpin family E member 2
SH-2	Src homology 2
SLN	Sentinel lymph node
TCR	T-cell receptor
TERF2IP	Telomeric repeat-binding factor 2-interacting protein 1
TERT	Telomerase reverse transcriptase
TGFBR1	Transforming growth factor beta receptor 1
Th	T helper cells
TIGIT	T-cell immunoreceptor with immunoglobulin and ITIM domain
TIM-3	T-cell immunoglobulin and mucin domain 3
TNF	Tumor necrosis factor
Treg	Regulatory T cell
TSC	Tuberous sclerosis complex
T-VEC	Talimogene laherparepvec
USA	United States of America
UV	Ultraviolet
VEGF	Vascular endothelial growth factor
